# Pedagogical Innovation in Digital Environments: A Systematic Review Focused on Higher Education

**DOI:** 10.12688/f1000research.176682.1

**Published:** 2026-04-15

**Authors:** Consuelo Nora Casimiro Urcos, Walther Hernan Casimiro Urcos, Nancy Elizabeth Alberca Pintado, Reynaldo Marcial Ostos Miraval, Miguel Alfredo Carrasco Muñoz, Livia Cristina Piñas Rivera

**Affiliations:** 1 National University of Education Enrique Guzman y Valle, Lima District, Lima Region, 15472, Peru; 2 National University of San Marcos, Lima District, Lima Region, 150101, Peru; 3 Uneval, National University Hermilio Valdizan, Huanuco, Huanuco, 10001, Peru

**Keywords:** Pedagogical innovation, digital environments, higher education, systematic review, digital competences.

## Abstract

This systematic review analyzed the scientific evidence published between 2020 and August 2025 on pedagogical innovations, digital environments, and university experiences. Its objective was to characterize how these categories have been investigated in terms of contributions, limitations, and results in higher education. Eligibility criteria were established that included studies (quantitative, qualitative, or mixed) developed exclusively in university contexts, with explicit methodological design, defined sample, peer review, and accessible data. Reviews, grey literature, articles without a specific sample or with unmitigated methodological bias were excluded. The sources of information were Scopus, Web of Science, ScienceDirect, Springer, SciELO, among others. The last search was carried out in August 2025, closing with the identification of 539 studies; of these, 39 met the inclusion criteria. Participants were university students, teachers, and experts (total = 9927) from different regions of the world, with sample sizes ranging from 7 to 3268; the average was 254.54. A validated instrument was applied to assess risk of bias, classifying studies as low risk (n = 22), moderate (n = 17), and high risk (n = 0). The results were synthesized through thematic analysis and tabulation of methodological characteristics. Improvements were reported in motivation, self-regulation, academic performance, and digital skills, although gaps in connectivity, teacher training, and institutional sustainability persisted. The results suggest that the impact of educational technology depends mainly on pedagogical design, teaching accompaniment and university policies.

## 1. Introduction

Higher education is going through a structural transformation where pedagogical innovation and digital environments operate inseparably. Thus, institutions have had to respond to the expansion of virtuality, the ubiquity of access to information and the demand for transversal skills; readjusting its curricular architecture and its teaching-learning frameworks. ^1^ In this transition, recent literature shows two complementary movements: on the one hand, the adoption of active methodologies supported by technologies that enhance student agency, collaboration, and meaningful learning ^2, 3^; on the other, the design of digital ecosystems –from LMS to laboratories and simulations– that enable experiences previously restricted to the classroom or physical laboratory. ^4, 5^ However, these advances confront tensions related to motivation, equity, data ethics and pedagogical sustainability. ^6– 8^

### Literature review

**Pedagogical innovations**

Active methodologies gain power when they are designed “with” and not only “in” technology, that is, when the digital resource is subordinated to clear pedagogical principles – authentic problematization, scaffolding, formative evaluation and *timely feedback.* Along these lines, Problem-Based Learning (PBL) supported by constructivist environments in the cloud raises creativity and digital skills if it articulates phases of activation, exploration, co-construction and transfer. ^3^ In postgraduate studies, for example, the PBL self-directed platform hybridization impacts procedural performance and mastery, as long as there are authentic tasks and times for guided reflection. ^9^ At the level of teacher training, the digital portfolio as the backbone of the *Personal Learning Environment* (PLE) favors self-regulation, traceability of progress and professional reflective thinking, ^10^ while flexible itineraries personalize routes according to rhythms, goals and evidence of learning. ^11^

Likewise, *Flipped Learning* shows consistent effects when it shifts transmission to the asynchronous, reserving the encounter for problem work, feedback, and metacognition, with gains in motivation and autonomy that are amplified with meaningful gamification. ^2, 12^ At the same time, online cooperative learning not only optimizes performance, but also critical socio-affective dimensions –sense of belonging, confidence in discipline, and reduction of academic loneliness–. ^13^ These improvements are not trivial: they are associated with design conditions such as positive interdependence, individual responsibility, and explicit rules of interaction.

In specific disciplinary areas, innovations combine authentic work and technology to maximize transfer. In engineering and construction, projects with *Building Information Modeling* (BIM) in university-business collaboration integrate sustainability, problem solving and employability. ^14^ In STEAM, the iterative incorporation of augmented reality (AR), 3D printing and GeoGebra forces continuous pedagogical redesigns and strengthening the adaptive capacity of teachers. ^15^ In language teaching, digitally mediated *Content and Language Integrated Learning* (CLIL) improves lexical-grammatical competence when accompanied by distributed practice and formative assessment.

Two complementary lines expand the scope of innovation. First, task-focused educational AI: the collaborative simplification of texts with a *Personal Learning Network* (NLP) improves comprehension and reduces barriers to access dense content if it is integrated with didactic criteria and *human feedback.* ^16^ Second, student-centered design: applying *Design Thinking* to create self-directed environments elevates digital literacy and performance, by aligning real problems, iteration, and evaluation with evidence. ^17^ This relocates the teacher as a designer of experiences and not only a user of platforms; a line that strengthens professional identity and pedagogical agency. ^18^ However, future teachers report gaps in empathy, curation, and digital self-presentation, dimensions that the curriculum must explicitly guide. ^19^ In a more critical key, emphasis is placed on humanizing digital pedagogy so that technology does not aggravate disconnections, but rather facilitates cognitive, social and teaching presence. ^20^

**Digital environments**

Environments have evolved from LMS ( *Learning Management System*) platforms to instructional ecosystems with simulation, virtual instrumentation, analytics, and collaboration layers. In health sciences, a web environment with theory, laboratory and virtual microscopy in parasitology increases self-efficacy and diagnostic transfer when progressively articulating cases and guided practice. ^5^ In geosciences, virtual microscopy based on open interactive resources expands inclusivity and accessibility without devaluing the disciplinary perceptual experience. ^4^ Virtual reality (VR) in the humanities makes it possible to “embody” immersion and situate interpretation in contexts that are otherwise unattainable, with effects on motivation and conceptual elaboration. ^21^ In engineering, asynchronous laboratory models demonstrate that complex practice can migrate to the digital environment if rigorous protocols, measurements, and feedback are ensured. ^22^

The adaptive potential of these ecosystems is evident when flexible itineraries and resource curation with pedagogical criteria are incorporated. ^11^ The AI-mediated layer of cognitive supports –for example, text simplification or homework tutors– is useful if it is integrated with transparency, clear boundaries, and teacher evaluation. ^16^ But not everything is technical: ethical design matters. Students perceive risks in their digital footprint –traceability, profiles and non-consensual uses –which requires data minimization policies, explicit purposes and informed consent. ^7^ Proposals such as *Online Learning as a Commons seek to* reverse the asymmetry of power, returning agency over data and rules of use. ^8^

The non-neutral nature of platforms deserves attention. From a socio-material perspective, *the Virtual Learning Environment* (VLE) configures teaching subjectivities (who speaks, how much, how it is monitored) and conditions of possibility of the class, generating openings, but also tensions –ritualizations of the *clickstream*, metrics that displace pedagogical judgment–. ^23^ Therefore, the focus of quality should not be “what platform”, but “what pedagogical-technical assembly” is produced: alignment of objectives, tasks, interactions and evidence; evaluation rules; and guarantees of inclusion, accessibility, and well-being.

**Higher education: experiences and results**

University experiences allow us to observe moderating variables and conditions of success. In science courses, *flipping* with guided activities and serious games increases motivation and autonomy if formative assessment is consistent. ^2^ In languages and culture, the combination of *flipped* and collaboration intensifies interaction and co-construction. In periods of abrupt transition (COVID-19), decreases in motivation and uneven success linked to connectivity and support gaps were observed, which reveals that the same digital device can produce opposite results depending on context, design and supports. ^6, 24^

In contrast, studies with diversified use of tools find increases in self-efficacy and achievement, especially when students have basic digital skills. ^25^ The management of the study also changes: digital note-taking in hybrid scenarios shows that agency, *affordances* and social norms mediate the adoption and perceived value of the tools. ^26^ At the level of curricula, integrating records and digital curation in archival opens up frontier professional competencies *blockchain*, forensics, preservation) if the redesign is intentional and evaluated. ^27^

Finally, experiences emerge that refine the innovation-evaluation relationship. In teacher training and health, virtuality-mediated PBL improves authentic problem design, pedagogical use of platforms and student performance, when accompanied by systematic training and pre-post measurement. ^28^ In English *as a Foreign Language* (EFL), LMSs (e.g., *Blackboard*) consolidate organization and follow-up, but their effect depends on the quality of interactions and *feedback.* ^29^ The cross-cutting lesson is clear: technology enables, but pedagogical design explains the results.

**Problematic situation**

Despite the systematized advances in this study, heterogeneities and gaps persist that justify the review. (i) Disparate results: positive effects on motivation, self-regulation, and achievement coexist with declines in motivation and performance in forced virtualization, moderated by age, level, and access. ^6, 11, 24, 25^ (ii) Asymmetries of access and competition: the effects depend on connectivity, resources and digital capital of students and teachers. ^23^ (iii) Ethics and governance: the massive adoption of analytics and traceability, without clear protocols, erodes the learner’s confidence and capacity, both to make conscious decisions and to plan, choose and take responsibility for their own learning. ^7, 8^ (iv) Pedagogical sustainability: short-term studies abound, longitudinal and comparative designs that estimate persistence of effects and transfer are lacking. ^3, 12^ (v) Disciplinary bias: health, education and languages concentrate evidence, while arts, humanities and social sciences are underrepresented, although VR and cultural projects point to promising paths. ^21, 30^ (vi) Professional identity: the shift of the teaching role towards the design of experiences is desirable, but it requires the development of curatorial competencies, social presence and ethics, which are not always addressed by training. ^18, 19^ (vii) Critical dimension: platforms configure practices and subjectivities, ignoring this mediation makes invisible tensions that affect quality and well-being. ^23^ In short, the field demands integrative frameworks that articulate instructional quality, inclusion, ethics, and evaluation with evidence.

This research sought to answer: What scientific evidence exists on pedagogical innovations, digital environments, and experiences and results in higher education, considering the analysis of their main contributions and limitations reported in the literature?

The general objective was to analyze the scientific evidence on pedagogical innovations, digital environments, and experiences and results in higher education, in order to characterize how these categories have been investigated based on the analysis of their main contributions and limitations reported in the literature.

## 2. Methodology

The review adhered to the methodological guidelines set out in the PRISMA 2020 Declaration, which provided the framework for transparently and rigorously planning, executing and reporting the processes linked to the analysis of scientific evidence on pedagogical innovations, digital environments, and experiences and outcomes in higher education. ^31^

### Eligibility criteria

Original studies published between 2020 and August 2025, exclusively in the field of higher education, which explicitly addressed some of the categories of analysis, were considered as inclusion criteria: pedagogical innovations, digital environments, or experiences and results in university contexts. Research with quantitative, qualitative and mixed approaches was considered, provided that in the latter case they complemented both methods and offered a complete and transparent methodological description. Only articles available in full text (in any language), published in indexed scientific journals with explicit peer review processes (double-blind or open) and full accessibility of data –including tables, figures and annexes– that would enable an exhaustive analysis and a thorough extraction of relevant information for the subsequent synthesis process were considered. Only texts with demonstrated quality were selected, assessed using risk of bias analysis tools used in this review, so as to endorse methodological rigor.

Duplicate studies (identified in the different search phases), systematic reviews, scoping reviews, and meta-analyses were excluded as they did not correspond to primary evidence. Purely exploratory original research was discarded, without developing analytical categories linked to pedagogical innovations, digital environments or experiences and results in higher education, as well as studies published outside the established period. Manuscripts from journals without a robust and verifiable peer review process were excluded, as well as research funded exclusively by companies with potential direct commercial conflict of interest, except in cases where the mechanisms in place to mitigate potential bias were explicitly stated. Sources classified as gray literature (web pages, general search engines, technical reports, institutional reports, theses or academic dissertations) were not considered, as they did not meet the criteria of rigor, accessibility and scientific validation required in this study.

### Sources of information

To identify the preliminary studies, an intensive and comprehensive search was carried out in various sources of information that potentially met the eligibility criteria, mainly databases and repositories of international scope such as Scopus, Web of Science, Springer, ScienceDirect, Scielo, and others.

According to Table 1, each source was consulted more than once to minimize the likelihood of bias and reinforce completeness. The search strategy was adapted to the characteristics of each database and its registration was carried out in the Mendeley bibliographic manager, selected for its effectiveness in the collection, organization and filtering of references, detection of duplicates and facilitation of collaborative work in the management of large volumes of information. ^32^

**Table 1.** Primary and complementary search strategies.

Main search	(TITLE (pedagogical innovation) AND TITLE (educational innovation)) AND (LIMIT-TO (DOCTYPE,"ar”)) AND (LIMIT-TO (OA,"all”)) (TITLE (digital learning) AND TITLE (digital environments)) AND PUBYEAR >2019 AND PUBYEAR <2026 AND (LIMIT-TO (DOCTYPE,"ar”)) AND (LIMIT-TO (OA,"all”)) (TITLE (digital pedagogy) AND TITLE (digital environments)) (TITLE (educational technology) AND TITLE (digital environments)) (TITLE (technology-enhanced learning) AND TITLE (digital environments)) (TITLE (online learning) AND TITLE (digital environments)) (TITLE (e-learning) AND TITLE (digital environments))
Additional searches	(TITLE(“pedagogical innovation”) OR TITLE(“teaching innovation”)) AND (TITLE(“higher education”) OR TITLE(“educational innovation”)) AND (LIMIT-TO (DOCTYPE,"ar”)) AND (LIMIT-TO (OA,"all”)) (TITLE(“innovative teaching”) AND TITLE(“digital transformation”)) AND PUBYEAR >2019 AND PUBYEAR <2026 AND (LIMIT-TO (DOCTYPE,"ar”)) (TITLE(“curriculum innovation”) AND TITLE(“digital environments”)) AND (LIMIT-TO (DOCTYPE,"ar”)) (TITLE(“blended learning”) AND TITLE(“digital environments”)) AND PUBYEAR >2020 AND PUBYEAR <2026 AND (LIMIT-TO (DOCTYPE,"ar”)) (TITLE(“virtual learning environments”) AND TITLE(“higher education”)) AND (LIMIT-TO (DOCTYPE,"ar”)) AND (LIMIT-TO (OA,"all”)) (TITLE(“adaptive learning”) AND TITLE(“digital environments”)) AND (LIMIT-TO (DOCTYPE,"ar”)) (TITLE(“artificial intelligence in education”) AND TITLE(“digital learning”)) AND (LIMIT-TO (DOCTYPE,"ar”)) AND (LIMIT-TO (OA,"all”)) (TITLE(“educational technology”) AND TITLE(“distance education”)) AND PUBYEAR >2020 AND PUBYEAR <2026 AND (LIMIT-TO (DOCTYPE,"ar”)) (TITLE(“digital pedagogy”) AND TITLE(“higher education”)) AND (LIMIT-TO (DOCTYPE,"ar”)) (TITLE(“MOOCs”) AND TITLE(“digital environments”)) AND PUBYEAR >2020 AND (LIMIT-TO (DOCTYPE,"ar”)) (TITLE(“gamification”) AND TITLE(“digital learning”)) AND (LIMIT-TO (DOCTYPE,"ar”)) AND (LIMIT-TO (OA,"all”)) (TITLE(“virtual reality”) AND TITLE(“education”)) AND PUBYEAR >2020 AND PUBYEAR <2026 AND (LIMIT-TO (DOCTYPE,"ar”)) (TITLE(“sustainable education”) AND TITLE(“digital environments”)) AND (LIMIT-TO (DOCTYPE,"ar”)) (TITLE(“inclusive education”) AND TITLE(“digital transformation”)) AND PUBYEAR >2020 AND (LIMIT-TO (DOCTYPE,"ar”)) (TITLE(“transformative learning”) AND TITLE(“digital innovation”)) AND (LIMIT-TO (DOCTYPE,"ar”))

### Study selection process

All potentially relevant records on pedagogical innovations, digital environments and experiences in higher education were processed. In the first phase, duplicates were identified and eliminated, and studies that did not meet eligibility criteria based on the title and abstract were discarded. Articles that passed this stage were reviewed in full text to confirm compliance with inclusion criteria. Finally, an additional critical review was carried out to minimize theoretical and methodological biases; the reasons for exclusion in this phase were documented in a specific table ( Table 3).

### Process for data extraction and risk of bias assessment

In this procedure, two researchers extracted data from quantitative studies following the methodological guidelines proposed by ^33^ in *Evaluating survey research in articles published in Library Science journals”.* The remaining authors extracted results from qualitative studies, applying the guidelines proposed by ^34^ in *“Validity criteria for qualitative research: three epistemological strands for the same purpose”.* The studies with a mixed design were evaluated by the whole team, combining both methodological proposals to ensure a coherent integration of evidence. Although it was planned to use a third evaluator to reach consensus in the event of discrepancies between reviewers regarding the data extracted, this was not necessary.

### Synthesis methods (tabulation and visual presentation of results)

The studies that met the eligibility criteria were organized in the table *“General characteristics of the included studies”* ( Table 2), indicating the total number of studies, Reference citation, country, potential source of bias, and general methodological information.

**Table 2.** General characteristics of the included studies.

No.	Reference citation	Country	Bias		General methodological information (Approach*** /Design**** /Participants*****)
			P *	D **	
1	^5^	United Kingdom and Spain	18	Low (L)	**E:** Quantitative. / **D:** descriptive and cross-sectional. / **P:** 95 Pharmacy students (4th year), Miguel Hernández University, Elche (Alicante, Spain).
2	^35^	Costa Rica and Spain	18	B	**E:** Quantitative. / **D:** Non-experimental, cross-sectional (ex post facto), analysis of personal learning environments (PLE). **Q:** 1187 university students, National University of Costa Rica.
3	^36^	Russia	17	Moderate (M)	**E:** Quantitative. / **D:** Quasi-experimental with cluster analysis. / **P:** 344 students (183 bachelor’s, 161 master’s, State University of Psychology and Education, Moscow.
4	^3^	Thailand	17	M	**E:** Mixed (qualitative and quantitative). / **D:** Design of development and validation of PBL model with constructivist environment in the cloud. / **P:** Panel of 7 experts in pedagogy and educational technologies.
5	^37^	Germany	16	M	**E:** Qualitative. **/D:** design of semi-structured interviews with qualitative content analysis (Mayring). / **P:** 12 STEM students (bachelor’s and master’s degrees), University of Lübeck, Germany.
6	^38^	Indonesia	18	L	**E:** Quantitative. / **D:** Non-experimental, correlational, with structural equation modeling (SEM). / **P:** 317 university students from the Faculty of Economic and Business Education, Universitas Pendidikan Indonesia (Bandung).
7	^2^	Morocco	18	L	**E:** Mixed. / **D**: Validation of instrument by means of a questionnaire (Likert, α Cronbach) applied to students; descriptive, ANOVA and correlational analysis. / **P:** 292 first-year students of Biological Sciences, Hassan II University of Casablanca (Morocco).
8	^4^	United Kingdom	18	L	**E:** Qualitative-descriptive with empirical validation. / **D:** Development of digital resource (Thinglink) with interactive microscopic images. / **P:** 26 people (6 students and 20 teachers), Geosciences programs at Keele University, United Kingdom.
9	^6^	Russia	17	M	**E:** Quantitative with qualitative complement. / **D:** Quasi-experimental and descriptive; application of surveys (Likert) and performance tests before and after three months of online teaching. / **P:** 600 adult students from five private language schools in Moscow, Russia (between 20 and 42 years old).
10	^16^	United Kingdom and Australia	18	L	**E:** Mixed. / **D:** Quasi-experimental with control/experimental groups and group focus analysis. **Q:** 46 university students (23 from *Education Studies* and 23 from *Digital Technology Solutions*), plus 2 professors at Manchester Metropolitan University (United Kingdom) and conceptual collaboration with RMIT (Australia).
11	^9^	Thailand	16	L	**E:** Quantitative-descriptive. / **D:** Course development with case-based learning (CBL) assessment in a digital self-learning environment (SDL) with quizzes and rubrics. / **P:** 14 students of Master’s Degree in Public Health, Mahidol University, Bangkok.
12	^14^	Spain and Portugal	17	L	**E:** Mixed. / **D:** Development of a sustainable industrial project with BIM methodology in a university-business collaborative environment. / **P:** 35 students of Industrial Engineering, University of Valladolid (Spain), with the collaboration of the University of Lisbon (Portugal) and the participation of companies from the automotive sector.
13	^11^	Spain	18	L	**E:** Qualitative. / **D:** Design-based research, developed in iterative cycles of analysis, development, implementation and evaluation of flexible digital itineraries. / **P:** 206 students of the Primary Education Teacher Category, University of the Balearic Islands.
14	^21^	USA	18	L	**E:** Mixed. / **D:** Case study with surveys of students and faculty. / **P:** 23 students (17 undergraduate, 6 graduate) and 12 professors from Lindenwood University (USA), in courses in Art History and Visual Culture.
15	^39^	Italy	17	M	**E:** Qualitative. / **D:** Case study in a university course of English Language and Culture; implementation of flipped classroom and cooperative learning in a digital environment. / **P:** 58 university students from the Università degli Studi di Milano (Italy).
16	^19^	Russia	18	L	**E:** Quantitative. / **D:** Descriptive-correlational with cluster analysis. / **P:** 200 students of Pedagogy (pre-service teachers), Herzen State Pedagogical University of Russia.
17	^24^	Mexico	17	M	**E:** Qualitative. / **D:** Exploratory-descriptive, with the application of questionnaires and analysis of teaching and student experiences during confinement. / **P:** 130 students and 10 teachers from the Autonomous University of San Luis Potosí, Mexico.
18	^40^	Portugal and France	17	M	**E:** Qualitative. / **D:** Pedagogical case study with three phases (documentary search, heuristic map and audiovisual pitch) integrated in a virtual collaborative environment (Padlet). / **P:** 18 students (10 from the Master’s Degree in Portuguese as a Foreign Language, Univ. do Minho; 8 from the Bachelor’s Degree in FLE, Univ. Bretagne Sud).
19	^25^	Saudi Arabia	18	L	**E:** Quantitative. / **D:** Descriptive, correlational, and comparative, with multiple regression and Rasch analysis for instrument validation. / **P:** 563 undergraduate students (talented and non-talented) from King Faisal University, Saudi Arabia.
20	^17^	Thailand	18	L	**E:** Mixed. / **D:** Design-based research, structured in iterative cycles, integrating *design thinking methodology.* / **P:** 10 experts in digital education and entrepreneurship, and 72 university students from King Mongkut’s University of Technology North Bangkok.
21	^7^	United Kingdom	17	M	**E:** Qualitative. / **D:** Inductive study with focus groups and reflective thematic analysis (Braun & Clarke). **Q:** 44 undergraduate students from three British universities, organised into 12 focus groups.
22	^41^	Spain	16	L	**E:** Mixed. / **D:** Single case study with online training intervention; analysis of asynchronous forums using *Epistemic Network Analysis* (ENA) and cluster analysis. / **P:** 13 students of a master’s degree in Distance Education, University of Seville (Spain).
23	^30^	Thailand	17	L	**E:** Qualitative. / **D:** Based on co-design and Kolb’s experiential learning model; iterative development of a digital platform. / **P:** 100 individuals, ages 25–44, including consumers and local textile artisans from Nakhon Si, Thailand.
24	^42^	Brazil	17	M	**E:** Quantitative. / **D:** Non-experimental, cross-sectional and exploratory. / **P:** 88 Pedagogy students from a public institution in the state of Pernambuco.
25	^43^	Russia	17	L	**E:** Quantitative. / **D:** Quasi-experimental with control and experimental group. / **P:** 42 university engineering students, Faculty of Energy, Samara State University.
26	^13^	Norway	16	M	**E:** Quantitative. / **D:** E interventionstudy, which compared the effect of digital cooperative learning (CA) and digital masterclasses on various psychosocial outcomes. / **Q:** Participants included 39 women (55%) and 32 men (45%), for a total of 71 participants.
27	^12^	Colombia	17	L	**E:** Quantitative. / **D:** Quasi-experimental, with pre-test and post-test in two groups. / **P:** 60 university students of Systems Engineering at the University of Magdalena, Santa Marta, Colombia.
28	^44^	Spain	18	L	**E**: Quantitative. / **D**: Descriptive study with a projectual approach in the classroom (PBL + flipped classroom). / **P**: 84 university students (3rd year), course in applied linguistics, University of Alicante (Spain)
29	^45^	Italy	19	L	**E:** Quantitative. / **D:** Quasi-experimental, with pre-test and post-test. / **P:** 99 first-year nursing students enrolled in the course “Pathophysiology Applied to Nursing” at a university in northern Italy.
30	^46^	China	16	M	**E:** Quantitative. / **D:** Experimental with pretest and posttest in two groups (RA vs. non-RA). / **P:** 28 university students from the Faculty of Education at the MARA Technical University.
31	^26^	United Kingdom	17	M	**E**: Mixed. / **D**: Explanatory sequential. / **P**: 123 students (various faculties, mostly undergraduate, 65% STEM), Focus groups: 17 students (various levels and disciplines, no representation of health sciences).
32	^22^	Vietnam	18	M	**E:** Quantitative. **/D:** experimental. / **P:** two groups of 200 students, including 100 in the control group (face-to-face practice) and 100 students in the experimental group (online feedback practice).
33	^47^	Kazakhstan	16	M	**E:** Quantitative. **/D:** Quasi-experimental. **/P:** 503 students enrolled in foreign language teaching programmes, distributed as follows: 250 full-time students; 255 distance education students.
34	^48^	Norway, Spain and Truquía	16	L	**E:** Qualitative. **/D:** Case study. **/P:** 86 university students (The course took place over 8 weeks).
35	^49^	Indonesia	18	L	**E:** Mixed. / **D:** pre-experimental with pre-test-post-test evaluations. / **P:** 120 students using the intentional sampling technique.
36	^50^	Indonesia	19	L	**E:** Quantitative. / **D:** Cross-sectional-Exploratory. / **P:** 385 Indonesian university students, analyzed using the structural equation modeling method.
37	^29^	Saudi Arabia	18	L	**E:** Mixed. / **D:** Sequential explanatory to explore students’ perceptions of educational technology. / **P:** 310 students, with varying levels of English proficiency.
38	^28^	Ecuador	19	L	**E:** Quantitative. **/D:** longitudinal quasi-experimental **. /P:** 120 mathematics teachers, enrolled in the Master’s Degree in Education in Digital Environments of the Bolivarian University of Ecuador and their respective students (3148 in total).
39	^23^	United Arab Emirates	16	M	**E:** Qualitative. / **D:** Phenomenological. /P: 7 teachers interviewed in human/digital contexts in a Virtual Learning Environment.

1 = Pedagogical innovations; 2 = Digital environments; 3 = Higher education – experiences/outcomes

* Score/

** Decision/Approach (E***) /Design (D****) /Participants (P****)

### Assessing bias in publication

To estimate the risk of bias derived from the absence of relevant results in the synthesis, differentiated procedures were applied, according to the type and volume of studies included. All the selected articles were evaluated using an instrument validated by experts, which considered the dimensions: title, abstract and keywords; introduction; literature review (explicit or implicit); methodology (materials, methods and ethical procedures); results; discussion; conclusions (contributions to knowledge and limitations); and bibliographic references. Each dimension was rated on an ordinal scale: Very Good (2 points), Good (1 point), Fair (0.5 points) and Deficient (0 points). The result of this evaluation yielded a vigesimal scale score (0 to 20), from which the level of risk of bias was estimated in three categories: low (18–20 points), moderate (15–17 points), and high (12–14 points). Studies with a score of less than 12 points were automatically excluded.

### Ethical considerations

At all times, the principles of academic integrity were respected and a rigorous citation was applied at each stage of the analysis, ensuring traceability and recognition of the original contributions.

## 3. Results

**Figure 1.**
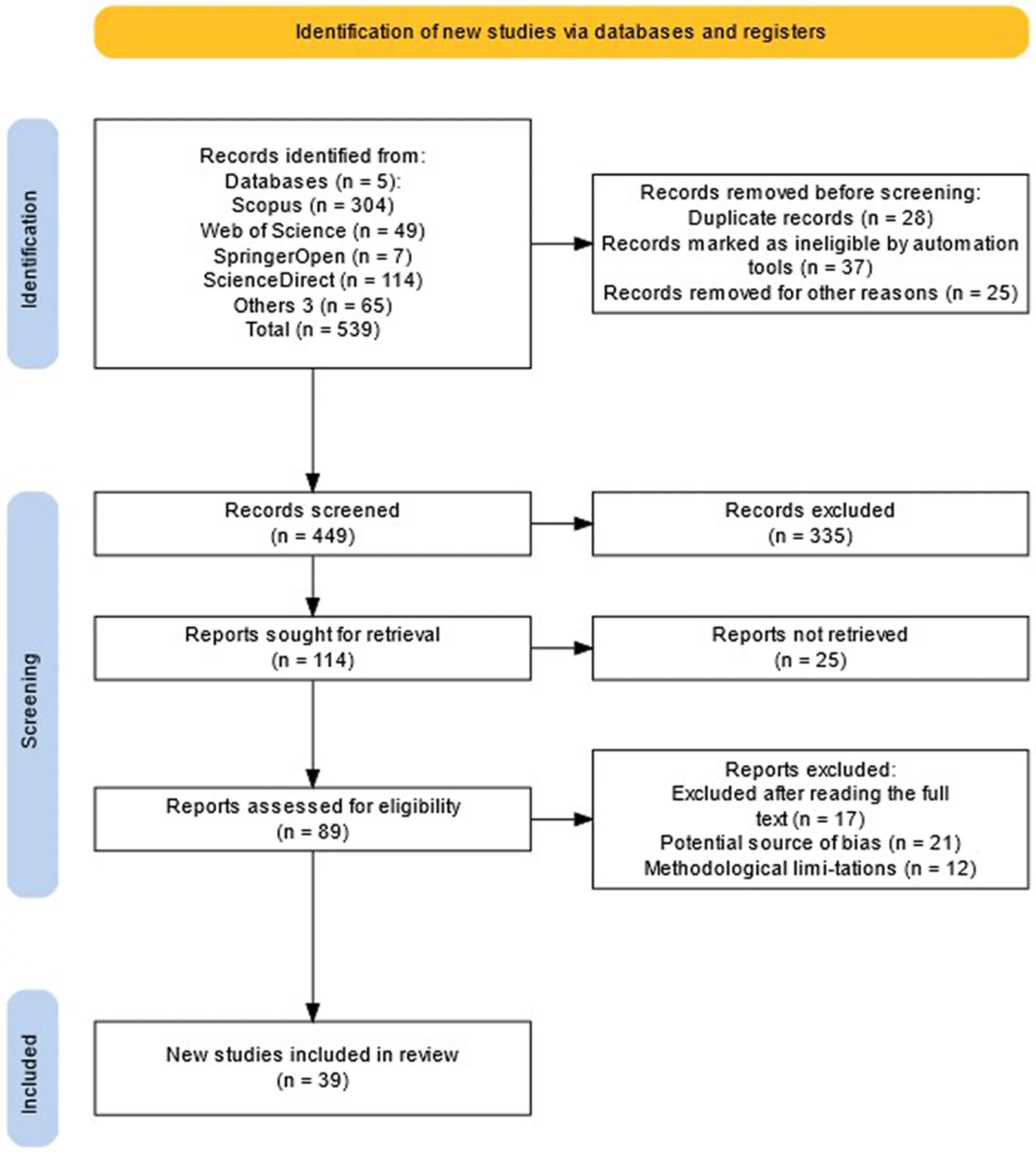
PRISMA 2020 flowchart.

Figure 1 identified 539 initial records from various databases and registries. Following the removal of duplicates and ineligible documents, 449 records were examined, of which 335 were excluded. Subsequently, 114 studies were considered for recovery, but 25 of them could not be obtained. Finally, 89 complete texts were evaluated, being excluded for the following reasons: potential bias (n = 21), methodological limitations (n = 12), non-compliance with eligibility criteria (n = 17). As a result, 39 studies were included in the final analysis.

Table 2 shows the inclusion of a total of 39 studies, with a cumulative sample of 9,927 participants and an average of 255 per research. In methodological terms, 10 qualitative (25.6%), 17 quantitative (43.6%) and 12 mixed (30.8%) studies were identified, reflecting a significant diversity of analytical approaches. In terms of origin, the countries of the United Kingdom (12.8%), Spain (12.8%), Thailand (10.3%) and Russia (7.7%) stand out; followed by Indonesia (7.7%), Saudi Arabia (5.1%), Portugal (5.1%), Italy (5.1%) and Norway (5.1%); other countries contributed 2.6% each, confirming the international nature of the research. Regarding the temporal distribution, production was concentrated in the last five years: 2020 with 3 studies (7.7%), 2021 with 6 (15.4%), 2022 with 9 (23.1%), 2023 with 7 (17.9%), 2024 with 8 (20.5%) and 2025 with 5 (12.8%). The most productive year was 2022, followed by 2024, which is evidence of a recent increase in research in pedagogical innovation and digital environments within higher education.

Table 3 shows valuable studies that, however, were excluded because they did not objectively specify population and sample, in addition to presenting methodological inconsistencies that limited their incorporation.

**Table 3.** Studies excluded after the last review process.

No.	Reference citation	Reasons for exclusion
1	^1^	Despite the quality of the findings, it was not possible to explicitly locate the methodological approach, and, within it, the population under study.
2	^8^	Despite its valuable conceptual approach to online learning as a common good and its theoretical contribution to the contemporary educational debate, it was excluded because it did not identify a specific population of study.
3	^15^	Although it presented valuable proposals for innovation in STEAM education and stands out for its integrative approach, when reviewing it in full text, it was not possible to confirm a detailed systematic methodology or specify the study population.
4	^27^	It provides relevant reflections on curricular updating in document management in the South African context and the 4IR. However, it was excluded for not defining a specific population
5	^51^	Although it presented clear, concise and well-explained results, it does not emphasize (or at least it was not possible to locate it in the full text) in a numerically defined population under study. Only stratified random sampling is mentioned, without indicating a final sample.
6	^52^	It mentions a clear methodology and the implementation of an experimental phase with diagnostic instruments such as the Mikhelson and Gilbukh test, interviews and Student’s t-test for adjacent groups; however, it does not specify the population or sample involved in the experimental phase. The exclusion is justified not by a lack of methodology, but by the absence of minimum population data required for rigorous systematic inclusion.

## 4. Discussion

This review analyzed scientific evidence on pedagogical innovations, digital environments, and experiences and results in higher education, in order to characterize how these categories have been investigated from the analysis of their contributions and limitations reported in the literature. To this end, 539 studies were identified, of which 39 met the inclusion criteria; in all cases closely linked to pedagogical innovation in digital environments in higher education, distributed in multiple regions of the world and with various methodological approaches (quantitative, qualitative and mixed). The most recurrent designs were quasi-experimental, case studies and correlational descriptive designs. Participants were university students, teachers and experts (total = 9927), with sample sizes ranging from 7 to 3268; the average was 254.54.

In the innovations, those with fundamental achievements in active participation, the development of autonomous learning and improvement of digital skills stood out; in these cases, they are specified through the incorporation of flipped classrooms, the use of interactive platforms and the use of methodologies based on design and experiential learning. Even when persistence of challenges in teacher training, the rejection of change and infrastructure were identified; In general, technological integration showed positive impacts on pedagogical innovation in higher education, by providing essential foundations for the analysis of limitations, implications, and perspectives of digital environments in this sector.

In this field of pedagogical innovation, its systematic implementation was essential for the profound rethinking –with a critical and creative orientation that transcends the superficial– of the conventional paradigms that support instruction and knowledge acquisition in the university environment; that is, both educational dynamics and training goals as well as functions of the actors involved. In this, there is a remarkable cohesion and redesign of didactic strategies –such as flipped classroom, gamification, PBL and experiential and collaborative learning– which, integrated into digital environments, optimize their applicability and performance. In addition to reconfiguring the traditional role of the teacher as transmitter, mediator and guide, the implementation of these strategies provides greater autonomy to students, empowering them as an active nucleus of the training process and promoting a meaningful, participatory and personalized assimilation of academic content. ^5, 11, 14, 35^

Furthermore, the implementation of hybrid pedagogical models, based on the articulation of active methodologies and virtual platforms, has favored the diversification of training scenarios in higher education. This diversification responds not only to technological demands, but also to emerging pedagogical and social needs, such as the flexibility of learning times and spaces, the inclusion of students with different profiles, and the incorporation of current competencies in academic programs. In addition to favoring access to knowledge, the face-to-face and virtual synergy of hybrid models has fostered transversal skills; for example, critical and collaborative thinking, self-management of learning and adaptability. For this reason, pedagogical innovation is conceived as a strategy aimed at enhancing the relevance, quality, and sustainability of the educational process in the contemporary university context. ^9, 25, 44^

In this framework, research underlines the potential of digital environments as mediators for the flexibility, contextualization and interactivity of training processes. Technological mediation that, in addition to redesigning traditional teaching-learning spaces, expands the methodologies used in terms of accessibility, active participation and multiplicity. In this, the systematic and core implementation in the articulation of specific subjects –not isolated or complementary– of LMS, AR resources, online collaborative tools, digital simulators, virtual learning objects, VR laboratories and multimedia materials was confirmed. Its implementation in several cases as a basic infrastructure for the comprehensive design of curricular programs has favored the expansion and geographical, social and cultural diversification of the scope of higher education. ^11, 16, 19, 23, 30^

Likewise, these studies underline the importance of adaptability and personalization in the design of virtual environments, through the use of developing technologies –AI, educational big data and learning analytics– as a dynamic and individual response to student requirements. ^43^ Furthermore, to positive effects on accessibility and equity, these proposals enhance essential elements for autonomy in learning (intrinsic motivation, self-regulation and sustained academic commitment). ^47^ More than repositories of content, digital environments act as catalysts for student self-management and the enhancement of metacognitive competencies vital for contemporary higher education, so that they function in the current pedagogical context as authentic complex ecosystems. ^22, 41, 46^

Furthermore, technological mediation in university processes is verified as a phenomenon of global scope, given the transversality of the category of higher education found in each of the materials included, as well as the multiplicity of university contexts represented. In addition to its globalization, this notes a concurrence of concerns that occupy the attention of higher education. These convergences point towards the necessary adaptation of pedagogical methodologies to the current training needs of a university student body with a critical spirit and a greater orientation towards virtuality and experiential learning. Consequently, environments that enhance interactivity, relevant curricular programs and strategies that situate academic content in order to solve specific problems are needed; that is, approaches with flexible models, which place the student in a central role of the training process. ^21, 24, 37^

In this sense, pedagogical innovation is more strongly needed in technical, scientific and professional profiles, where the management of emerging technologies has had a significant impact on the expansion of academic options. Today’s students have the opportunity to strengthen the theory-practice link through technologies that allow them to simulate, experiment and represent phenomena in complex environments; while promoting the deep apprehension of training content, they also develop skills demanded by the labor market. ^28, 38, 45^

Moreover, to the assimilation of virtual tools, in the university context these innovations promote a complex process of comprehensive reformulation that enhances a training ecosystem of greater coherence and reflexibility, focused on the student. Essential dimensions such as instructional design, formative assessment, and the teacher’s mediating accompaniment are involved in this empowerment. From a constructive orientation, it is seen how these innovations are interested in a rigorous and conscious alignment of training with pedagogical strategies and evaluation components, which favors the development of specific competencies and the acquisition of deep, transmittable and lasting learning. ^7, 26^

The results confirm that pedagogical innovations in digital environments demand conditions that go beyond the mere will of teachers, while highlighting the importance of their continuous professional development, oriented towards technical skills and the formative apprehension of technology. They also agree on the need to implement sustained university policies that stimulate continuous training and counseling programs, as well as the creation of environments for pedagogical experimentation and feedback. This institutional vision of innovation allows for the consolidation of a culture of continuous improvement, where technologies are not an end, but a means to promote more relevant, inclusive and contextualized pedagogical practices. ^4, 23, 40^

From the methodological point of view, these studies present a remarkable diversity of approaches and designs, which enriches the understanding of phenomena linked to pedagogical innovation in digital environments within higher education. This methodological variety not only reflects the progressive maturity of the field, but responds to the multifaceted and contextualized nature of technology-driven educational transformation processes. Research developed under quantitative, qualitative and mixed approaches is identified, encompassing experimental, quasi-experimental, exploratory, descriptive designs, case studies and design-based research. This variety, more than a limitation, corroborates the complexity of educational innovation practices and makes it possible to analyze their impacts, meanings and contradictions from different positions and epistemological points of view. ^13, 36, 39, 42^

The strength of the results is also supported by highly complex and robust analysis techniques –from the statistical or qualitative point of view– of some of these studies. In addition to contributing to analytical soundness, these techniques promote the establishment of explanatory and relevant relationships between the variables studied, the detection of emerging patterns and the understanding of dynamic interactions present in the teaching-learning processes with technological mediation. Overall, the analysis of epistemic networks, the modeling of structural equations, focus groups and clusters stand out. The plurality and rigor of this methodological approach strengthen the internal/external validity of these investigations, providing a holistic vision on the construction, implementation and evaluation of pedagogical innovation in the contemporary field of higher education. ^20, 25, 53^

In this systematic review, the findings confirm a positive and consistent succession of effects on various variables linked to technology-mediated university education. These include motivation, commitment, satisfaction with the training process, academic performance and assimilation of key competencies; on the digital, communicative, collaborative and metacognitive level. These results corroborate that the strategic integration of pedagogical innovations in digital environments has the potential to significantly and sustainably transform the experience of university education; overcoming the traditional one-way transmission of knowledge. However, studies underline the need to design and implement these innovations from rigorous standards of quality, relevance, contextualization and institutional sustainability as the only way to avoid superficiality in the technification of pedagogical processes. ^14, 17, 50^

Over and above that, to the individual benefits of learning, several studies highlight the structural potential of these experiences to promote fundamental principles such as equity, inclusion, and universal access to higher education, especially in historically vulnerable, lagging, or isolated populations. Technological mediation is revealed as a powerful tool to democratize educational opportunities, expand spaces for participation and overcome physical, economic or cultural barriers that limited the educational trajectory of certain groups. This inclusive and transformative approach reinforces the role of universities not only as centres of knowledge, but as active agents in the construction of a fairer society with higher levels of social cohesion. ^2, 12, 48^

Some studies also report no minor limitations, tensions and structural challenges associated with the implementation of pedagogical innovations in digital environments within higher education. Difficulties that show that the processes of educational transformation do not always develop in ideal conditions. Among the most frequent obstacles are resistance to change on the part of some teachers –who may perceive innovations as a threat to their consolidated practices– deficient technological infrastructure, little training in digital skills, cognitive overload generated by the simultaneous or excessive use of platforms, as well as the urgency of guaranteeing accessibility, usability and pedagogical quality in the digital resources used. ^3, 6, 29^ These limitations call for rethinking innovation not as a mere technological addition, but as a systemic and integral process that requires the coherent articulation of pedagogical, technological, organizational, ethical, and formative dimensions of university practice.

## 5. Conclusions

In the pedagogical innovations, it was found that strategies such as the flipped classroom, problem-based learning, gamification, experiential learning and collaborative learning had been adapted to digital environments, generating positive effects on motivation, participation and development of transversal competences. These practices showed commitment to the promotion of active and transferable learning, in pursuit of overcoming the traditional unidirectional models of transmission. However, the contributions were conditioned by the coherence of objectives-strategies-evaluation, as well as by teaching responsibility and institutional support. Among the main limitations were perceived insufficiencies in the training of digital skills, rejection of change and lack of policies aimed at the sustainability of these practices.

As for digital environments, their evolution from mere information receptacles to complex ecosystems of positive impacts on accessibility, flexibility and personalization of learning was confirmed; effects that favor, in turn, student autonomy and self-regulation. However, considerable challenges were identified: disparity in access to connectivity, cognitive overload derived from the prolonged use of platforms, usability problems in some resources and ethical dilemmas on data management. Beyond the technological component *per se*, the gain of these environments lay in their integration with a pedagogical design oriented towards the humanization, inclusiveness and contextualization of education.

In the higher education category, the analysis of experiences and results showed a favorable influence on student motivation and satisfaction, academic performance and the acquisition of competencies in the digital, communicative, collaborative and metacognitive planes. A relevant transformation was found in the university educational experience associated with the integration of pedagogical innovations and digital tools, as long as they were implemented under criteria of quality and relevance. However, its effectiveness showed disparate effects –even counterproductive– in scenarios of abrupt transition or communities with insufficient technological resources. This disparity highlighted the essentiality of the pedagogical strategy, the mediating role of the teacher and the institutional support for obtaining favorable results, beyond technological accessibility.

Overall, this review found that pedagogical innovation mediated by digital environments should be considered a strategic instrument for the reconfiguration of higher education towards transformative paradigms of greater humanism and inclusiveness; not as an objective *per se.* These advances indicated a strengthening of the quality of training and a gain in equitable access to knowledge, in those institutions that achieved synergistic integration between pedagogical and technological foundations. The urgency of subordinating technology to educational purposes was also highlighted, overcoming instrumental paradigms and maintaining the human relationship as a nuclear element.

## Contribution to knowledge and future lines of work

These findings could favor the understanding of the transformative role of pedagogical innovations and digital environments in the university training context, provided that their articulation is guaranteed from coherent pedagogical designs, with robust institutional policies and equitable access to education. The knowledge provided could be a guide in terms of inclusivity and sustainability, as well as in the identification of limitations for its impact on higher education. It is advisable to promote interdisciplinary comparative research aimed at exposing nuances in technological assimilation, as well as longitudinal studies aimed at assessing the permanence of the observed impacts. Likewise, critical studies on data analytics and AI are recommended as new routes of exploration based on humanism and equity. Finally, exploration in areas of social sciences and humanities could be valuable, given that the possibilities offered by the incorporation of digital environments in these areas are still insufficiently explored.

## Data Availability

Zenodo: Pedagogical Innovation in Digital Environments: A Systematic Review Focused on Higher Education: https://doi.org/10.5281/zenodo.10799108. ^54^ This project contains the following data:
• 01 PRISMA_2020_checklist.pdf • PRISMA Flow chart • Complete list of the included studies Data are available under the terms of the Legal Code - Attribution-ShareAlike 4.0 International - Creative Commons (CC BY-SA 4.0).
