# Supplementary material for: Pedagogical Innovation in Digital Environments: A Systematic Review Focused on Higher Education
Source: F1000Res. 2026 Apr 15;15:522. [Version 1] doi: 10.12688/f1000research.176682.1 (PMC13570119; doi:10.12688/f1000research.176682.1)
Supplement: Peer review history

# Reviewer report: Reviewer response for version 1

**DOI:** 10.5256/f1000research.194769.r480747

**Published:** 2026-05-15

**Authors:** Enrique Renteria Castro

**Affiliations:** 1 National Association of Universities and Higher Education Institutions, Mexico City, Mexico

The review carried out by this article is very important; it manages to make a systematic description of the use of digital tools in higher education and how they have been incorporated into teaching practice as innovative strategies of pedagogical practice.

 With respect to the problematic situation, the review of each of the articles that contribute to defining educational innovation with the use of digital tools allows us to reflect on sustainable development in higher education when using technological advances, including artificial intelligence. The same studies analyzed show that the lack of infrastructure for the use of digital technologies and the absence of didactic-pedagogical training for teachers are immediate references to the limitations to implement innovative educational actions.

 With respect to the results of the conclusions, I consider that the studies reviewed give evidence that different authors have discarded the conceptual approach to didactics and pedagogical models. They should consider that in humanities and social sciences careers, the design of curricular projects includes a classification of subjects in correspondence with a certain pedagogical model; for example, there are monographic courses, workshops, seminars, professional practices, among others. In the case of monographic courses, the use of the frontal transmission of knowledge has been defined as the art of mastering communicative processes and that even this pedagogical model has been impacted with pedagogical innovations with the use of digital tools. As an example, we can refer to videos, detonating questions for forum work, Socratic maieutics as an innovative strategy and without detracting from the traditional pedagogical model (I use the concept of pedagogical model as opposed to the concept of you: pedagogical agency). In the case of seminars defined in the social sciences as the learning laboratory proposed in the same way as laboratories in the natural sciences (J. Gaos 1956), activities such as analysis of texts, summaries, videos, exhibitions, interpretations, debates and research are carried out and, of course, today using the internet, digital tools and AI, which allows an extensive approach to research and documentary materials that allow defining, framing and solving problems in the social and humanistic disciplines.

 Pedagogical innovation should not only be seen as a need to update the technical, scientific and professional profile of a teacher; we must consider that the teacher has moved from a fluctuating dynamic between empirical and isolated practice, which is perfected with experience, to being considered as a practice intimately articulated to intellectual processes. Today, in higher education systems aligned with virtuality and with the use of digital tools, the teacher must propose to move towards the conceptualization of group, interdisciplinary and collegial work, to transform his individual practice towards socio-interactive teaching, which goes beyond one-person activities and the simple transmission of knowledge. Socio-interactive teaching must not only impact an educational institution but also the governance of the local educational systemal, national and international.

 In 1997, the first LMS appeared for generic use in educational institutions, but it was not until 2020 with COVID-19 that higher education teachers, pressured by confinement, explored the use of digital tools, generating an improvisation and decontextualization of didactic and pedagogical principles, which you point out in your research: To design a systemic humanistic approach that seeks inclusion, equity and well-being.

 Not only the flipped classroom, problem-based learning, gamification, experiential learning and collaborative learning are the only didactic strategies that allow students to mobilize towards participatory and transformative learning, we must consider and rescue the contributions of group theory (technical and dynamic), verbal and non-verbal communication and the different pedagogical models that allow us to define a conceptual framework to use technology as a means and achieve an education based on teaching and learning for transformative participation.

 I have a doubt: will it be convenient to use the phrase: development of key competences, instead of assimilation of key competences.

 Molina, E., & Medina, E. (2025). La revolución de la IA en la Educación Superior. Lo que hay que saber. En Innovaciones Digitales en Educación. Banco Mundial.

Are the rationale for, and objectives of, the Systematic Review clearly stated?

Yes

Is the statistical analysis and its interpretation appropriate?

Yes

If this is a Living Systematic Review, is the ‘living’ method appropriate and is the search schedule clearly defined and justified? (‘Living Systematic Review’ or a variation of this term should be included in the title.)

Yes

Are sufficient details of the methods and analysis provided to allow replication by others?

Yes

Are the conclusions drawn adequately supported by the results presented in the review?

Yes

Reviewer Expertise:

Curricular design and evaluation, consulting for accreditation of higher education study plans to obtain RVOE. Founding member of the National Network for Peace of the National Association of Universities and Higher Education Institutions. International Evaluator of Higher Education Quality, accredited by UDUAL and CIIES. Director General of Evaluation and Educational Development. Academic of doctoral programs at the Global Latin American University and at UNAM.

I confirm that I have read this submission and believe that I have an appropriate level of expertise to confirm that it is of an acceptable scientific standard.

## References

[1] : Deslinde conceptual entre educación en línea o educación a distancia. *Delectus* .2021;4(1) : 10.36996/delectus.v4i1.9816-31 10.36996/delectus.v4i1.98

[2] : An analysis of the online information searching strategies and metacognitive skills exhibited by university students during argumentation activities. *Library & Information Science Research* .2020;42(3) : 10.1016/j.lisr.2020.101019 10.1016/j.lisr.2020.101019

# Reviewer report: Reviewer response for version 1

**DOI:** 10.5256/f1000research.194769.r476284

**Published:** 2026-05-12

**Authors:** Anass Bayaga

**Affiliations:** 1 Stellenbosch University, Stellenbosch, South Africa

**Recommended status: Not Approved**

 The manuscript addresses a relevant topic in higher education, namely pedagogical innovation in digital environments. The review reports that 539 records were identified and 39 studies were included, covering issues such as motivation, self-regulation, academic performance, digital skills, connectivity, teacher training and institutional sustainability. However, the paper requires substantial revision before it can be considered methodologically sound.

 The main concern is that the manuscript is poorly written in several places, and this affects meaning and readability. For example, phrases such as “pedagogical innovation and digital environments operate inseparably”, “readjusting its curricular architecture”, “formative apprehension of technology”, and “human relationship as a nuclear element” are unclear or awkward. The paper needs professional academic language editing, not only minor proofreading.

 The methodology also needs strengthening. Although the authors state that they followed PRISMA 2020, the search strategy is not sufficiently reproducible. Many searches appear to rely on title-only terms such as “pedagogical innovation” and “digital environments”. This may have excluded relevant studies that discuss these concepts in the abstract or keywords but not in the title. The authors should provide the full search string for each database, including dates searched, filters applied, and justification for the title-only approach.

 The risk-of-bias assessment is also unclear. The authors report that 22 studies were low risk, 17 moderate risk and none high risk. However, the tool appears to assess general article quality, including title, abstract, introduction, literature review and references, rather than methodological bias. This distinction is important. The authors should either rename this as a quality appraisal or use recognised design-appropriate appraisal tools for quantitative, qualitative and mixed-methods studies.

 The synthesis is too descriptive. The paper states that thematic analysis was used, but it does not explain how themes were generated, how studies were coded, or how evidence from different designs was integrated. The three categories — pedagogical innovation, digital environments and higher education experiences/results — need clearer analytical justification.

 Some conclusions are too broad. For example, the claim that digital pedagogical innovation can reconfigure higher education towards “greater humanism and inclusiveness” is conceptually interesting but not sufficiently supported by the evidence presented. The conclusions should be more cautious and directly linked to the findings.

 Overall, the manuscript has potential, but it requires major revision. The authors should improve the language, clarify the search and screening process, justify the appraisal tool, strengthen the synthesis, revise Table 2 for readability, and ensure that the conclusions are proportionate to the evidence.

Are the rationale for, and objectives of, the Systematic Review clearly stated?

No

Is the statistical analysis and its interpretation appropriate?

No

If this is a Living Systematic Review, is the ‘living’ method appropriate and is the search schedule clearly defined and justified? (‘Living Systematic Review’ or a variation of this term should be included in the title.)

No

Are sufficient details of the methods and analysis provided to allow replication by others?

No

Are the conclusions drawn adequately supported by the results presented in the review?

No

Reviewer Expertise:

Mathematics Cognition: Neuro-Mathematics, STEM Cognitive Enhancement and Human-Computer Interaction

I confirm that I have read this submission and believe that I have an appropriate level of expertise to state that I do not consider it to be of an acceptable scientific standard, for reasons outlined above.

# Reviewer report: Reviewer response for version 1

**DOI:** 10.5256/f1000research.194769.r480741

**Published:** 2026-05-08

**Authors:** Andrea Basantes-Andrade

**Affiliations:** 1 Fecyt, Universidad Técnica del Norte, Ibarra, Imbabura Ecuador, Ecuador

The manuscript addresses a relevant and contemporary topic in higher education, focusing on pedagogical innovation in digital environments and its implications for university teaching and learning processes. The systematic review gathers recent evidence published between 2020 and 2025, incorporating studies from different international contexts and diverse methodological approaches. The article presents a solid introduction, with an appropriate contextualization of the problem, a clear identification of research gaps, and an explicit formulation of the research question and objective. Likewise, the authors’ effort to integrate pedagogical, technological, and educational perspectives within a broad and current analytical framework is acknowledged.

 However, although the topic is relevant and the manuscript has scientific potential, there are important methodological and reporting limitations that must be addressed to ensure the rigor, transparency, and reproducibility expected in a systematic review aligned with PRISMA 2020 and the standards of F1000Research.

 First, the documentary search strategy requires greater precision and methodological transparency. The manuscript presents only partial fragments of the search equations and does not report the complete search strategies for each database consulted. Furthermore, the exact search dates for each database, language filters, operators used on each platform, and the use of controlled vocabulary or specialized thesauri are not clearly specified. This lack of detail limits the reproducibility of the study. It is recommended that the authors include, in an appendix, all complete and detailed search strategies, indicating the specific search dates and justifying the methodological decision to restrict the search exclusively to titles (“TITLE”).

 Regarding the study selection process, the manuscript does not sufficiently explain how the article screening process was conducted. Although duplicate removal and phased review procedures are mentioned, the manuscript does not specify how many reviewers participated in the process, whether the evaluations were conducted independently, or how disagreements between reviewers were resolved. Likewise, no inter-rater agreement indices, such as Cohen’s Kappa coefficient or equivalent measures, are reported. These elements are fundamental to ensuring methodological consistency and reducing selection bias. Therefore, it is recommended that the authors describe in detail the review and consensus procedures followed among reviewers.

 Another aspect that requires strengthening is the data extraction process. The manuscript states that different researchers analyzed quantitative and qualitative studies using different methodological guidelines; however, no extraction matrix is presented, nor are the extracted variables, analytical categories, or coding procedures clearly specified. It is suggested that the authors incorporate a table or methodological appendix explicitly describing the variables collected from each study, the categorization criteria, and the procedures used to handle ambiguous or incomplete information.

 One of the main methodological weaknesses of the article concerns the assessment of risk of bias. The authors used a self-developed instrument based mainly on criteria related to article structure and writing quality, rather than on a robust methodological evaluation of the risk of bias of the included studies. Although the instrument appears organized, it has not been internationally validated, and no evidence of reliability or justification for the cutoff points used is reported.

 Regarding the analysis and synthesis of results, although the review develops a coherent and thematically well-structured narrative synthesis, the absence of a meta-analysis is not adequately justified, nor is it clearly explained how the thematic categories used in the discussion were constructed. The manuscript reports multiple positive effects on motivation, performance, and digital competencies; however, these findings are not accompanied by effect measures, heterogeneity analyses, or systematic comparisons between studies. The authors are encouraged to explicitly justify the choice of a narrative synthesis, explain the thematic analysis procedures, and discuss more deeply the methodological and contextual heterogeneity of the included studies.

 In addition, several inconsistencies were identified in the presentation of numbers, percentages, and methodological categories in tables and figures, which should be carefully reviewed to improve the technical accuracy of the manuscript. In Table 2, for example, there are contradictions between the assigned scores and the classification of risk of bias. According to the methodology section, scores between 18–20 correspond to low risk, whereas scores between 15–17 correspond to moderate risk; however, some studies with a score of 16 are classified as “Low (L),” which contradicts the criteria defined by the authors themselves. Likewise, inconsistencies exist in the abbreviations used for participants (“P:” and “Q:”) without a clear methodological explanation.

 Minor typographical errors and formatting issues were also observed in the table, such as incomplete expressions or incorrect word separations, possibly resulting from document conversion problems. Although the overall percentages reported in the methodological distribution are mathematically correct, it is recommended to standardize the use of decimal places and review the statistical consistency among the text, tables, and figures.

 In the PRISMA figure, the reported values are generally coherent; however, the visual presentation of the exclusion reasons makes it difficult to quickly interpret the methodological flow. Improving the readability of the diagram and organizing the exclusion categories more clearly is recommended. Similarly, Table 3 presents only individual examples of excluded studies but does not incorporate a systematic classification or overall counts by exclusion reason, which limits the methodological traceability of the review.

 On the other hand, the conclusions tend in some sections to overgeneralize the findings. The repeated use of expressions such as “confirmed,” “demonstrated,” or “generated positive effects” may be excessively deterministic considering that a large proportion of the included studies are descriptive, qualitative, or quasi-experimental in design. It is recommended that the authors adopt more cautious language consistent with the level of available evidence, using expressions such as “suggests,” “indicates,” or “is associated with.”

 It would also be important to strengthen the critical discussion regarding the structural and ethical limitations of digital environments. Although the manuscript mentions issues related to connectivity and teacher training, topics such as digital inequality, algorithmic surveillance, platform bias, institutional sustainability, and risks associated with the growing use of artificial intelligence in higher education could be explored in greater depth. A more critical perspective would enrich the scientific contribution of the article.

 I encourage the authors to address the issues identified above. Before the manuscript can be considered scientifically robust and suitable for indexing, substantial methodological revisions related to transparency and reproducibility are required. With these improvements, the work could represent a valuable contribution to the field of pedagogical innovation and digital environments in higher education.

Are the rationale for, and objectives of, the Systematic Review clearly stated?

Yes

Is the statistical analysis and its interpretation appropriate?

Partly

If this is a Living Systematic Review, is the ‘living’ method appropriate and is the search schedule clearly defined and justified? (‘Living Systematic Review’ or a variation of this term should be included in the title.)

Not applicable

Are sufficient details of the methods and analysis provided to allow replication by others?

Partly

Are the conclusions drawn adequately supported by the results presented in the review?

Partly

Reviewer Expertise:

Educational Technology, Digital Education, Higher Education, ICT in Education, E-learning, Digital Competencies, Artificial Intelligence in Education, Inclusive Education, Systematic Reviews, Educational Innovation.

I confirm that I have read this submission and believe that I have an appropriate level of expertise to confirm that it is of an acceptable scientific standard, however I have significant reservations, as outlined above.

## References

[1] : The PRISMA 2020 statement: an updated guideline for reporting systematic reviews. *BMJ* .2021; 10.1136/bmj.n71 10.1136/bmj.n71 PMC800592433782057
